# Point-Of-Care low-field MRI in acute Stroke (POCS): protocol for a multicentric prospective open-label study evaluating diagnostic accuracy

**DOI:** 10.1136/bmjopen-2023-075614

**Published:** 2024-01-31

**Authors:** Matteo Foschi, Angelo Galante, Raffaele Ornello, Stefano Necozione, Carmine Marini, Mario Muselli, Paola Olimpia Achard, Luciano Fratocchi, Sergio Lucio Vinci, Marco Cavallaro, Mauro Silvestrini, Gabriele Polonara, Simona Marcheselli, Laura Straffi, Marco Colasurdo, Luca Sorrentino, Enrico Franconi, Marcello Alecci, Massimo Caulo, Simona Sacco

**Affiliations:** 1 Department of Biotechnological and Applied Clinical Sciences, University of L'Aquila, L'Aquila, Italy; 2 Department of Life, Health & Environmental Sciences, University of L'Aquila, L'Aquila, Italy; 3 National Institute for Nuclear Physics, Gran Sasso National Laboratory, L'Aquila, Italy; 4 SPIN-CNR, c/o Department of Physical and Chemical Science, University of L'Aquila, L'Aquila, Italy; 5 Department of Industrial and Information Engineering and Economics, University of L'Aquila, L'Aquila, Italy; 6 Department of Biomorf, University of Messina, UOC Neuroradiology, Messina, Italy; 7 Department of Experimental and Clinical Medicine, Neurological Clinic, Marche Polytechnic University, Ancona, Italy; 8 Department of Odontostomatological and Specialized Clinical Sciences, Polytechnic University of Marche, Ancona, Italy; 9 Emergency Neurology and Stroke Unit, IRCCS Humanitas Research Hospital, Rozzano, Italy; 10 Department of Neuroscience and Clinical Sciences, University of Chieti, Chieti, Italy; 11 Department of Civil and Mechanical Engineering, University of Cassino and Southern Lazio, Cassino, Italy; 12 Faculty of Computer Science, Free University of Bozen-Bolzano, Bolzano, Italy

**Keywords:** Stroke, Diagnostic Imaging, Magnetic resonance imaging

## Abstract

**Abstract:**

**Introduction:**

Fast and accurate diagnosis of acute stroke is crucial to timely initiate reperfusion therapies. Conventional high-field (HF) MRI yields the highest accuracy in discriminating early ischaemia from haemorrhages and mimics. Rapid access to HF-MRI is often limited by contraindications or unavailability. Low-field (LF) MRI (<0.5T) can detect several types of brain injury, including ischaemic and haemorrhagic stroke. Implementing LF-MRI in acute stroke care may offer several advantages, including extended applicability, increased safety, faster administration, reduced staffing and costs. This multicentric prospective open-label trial aims to evaluate the diagnostic accuracy of LF-MRI, as a tool to guide treatment decision in acute stroke.

**Methods and analysis:**

Consecutive patients accessing the emergency department with suspected stroke dispatch will be recruited at three Italian study units: Azienda Sanitaria Locale (ASL) Abruzzo 1 and 2, Istituto di Ricerca e Cura a Carattere Scientifico (IRCCS) Humanitas Research Hospital. The estimated sample size is 300 patients. Anonymised clinical and LF-MRI data, along with conventional neuroimaging data, will be independently assessed by two external units: Marche Polytechnic University and ‘G. Martino’ Polyclinic University Hospital. Both units will independently adjudicate the best treatment option, while the latter will provide historical HF-MRI data to develop artificial intelligence algorithms for LF-MRI images interpretation (Free University of Bozen-Bolzano). Agreement with conventional neuroimaging will be evaluated at different time points: hyperacute, acute (24 hours), subacute (72 hours), at discharge and chronic (4 weeks). Further investigations will include feasibility study to develop a mobile stroke unit equipped with LF-MRI and cost-effectiveness analysis. This trial will provide necessary data to validate the use of LF-MRI in acute stroke care.

**Ethics and dissemination:**

The study was approved by the Research Ethics Committee of the Abruzzo Region (CEtRA) on 11 May 2023 (approval code: richyvgrg). Results will be disseminated in peer-reviewed journals and presented in academic conferences.

**Trial registration number:**

NCT05816213; Pre-Results.

**STRENGTHS AND LIMITATIONS OF THIS STUDY**

- This is the first study evaluating the diagnostic accuracy of low-field (LF) MRI in acute stroke care, as a tool to guide treatment decisions.
- The multicentric design will allow to investigate the diagnostic performance of LF-MRI both on site and independently by external units.
- The performance of LF-MRI will be evaluated at different time points, providing information on the evolution of stroke lesions over time (24 hours to 4 weeks).
- Further investigations will include cost-effectiveness analysis and feasibility study to develop an ambulance equipped with LF-MRI.
- Study limitations include its non-randomised design, although clinical and LF-MRI data will be compared with conventional neuroimaging findings in a blinded fashion.

## Introduction

 Stroke is the second-leading cause of mortality and morbidity worldwide, resulting in more than 5.5 deaths and over 110 million disability-adjusted life-years lost per year.^1^ The annual incidence rates in the Italian population ranges from 175:100 000 to 360:100 000 in men and from 130:100 000 to 273:100 000 in women.^2^ Stroke can be broadly classified into ischaemic and haemorrhagic, the latter of which include intracerebral and subarachnoid haemorrhage. Ischaemic stroke refers to an infarction of the brain, spinal cord or retina and accounts for ~71% of all strokes globally.^3^ Advances in brain imaging identified that much of the initial clinical deficit in patients with stroke is related to a hypoperfused non-functional part of the brain termed ischaemic penumbra. This region progressively evolves to irreversibly injured tissue over time (ischaemic core).^3^ However, rapid reperfusion can salvage the ischaemic penumbra leading to restoration of normal function. This discovery formed the rationale for reperfusion therapies, which are strictly time dependent. Intravenous thrombolysis (IVT) can reduce the disability when administrated within 4.5 hours of stroke onset (defined as the time from when the patient was last known to be healthy), although selected patients with favourable brain perfusion imaging may benefit up to 9 hours.^4^ Endovascular thrombectomy (EVT) reduces disability in patients with cerebral large vessel occlusion (LVO) through the mechanical retrieval of the clot via catheter angiography. EVT must be performed within 6 hours of the time the patient with a stroke was last to known to be healthy and, in patients selected using brain perfusion imaging, up to 24 hours.^5^ The outcome of both IVT and EVT relies on the accuracy of diagnosis and on the time from symptoms onset to treatment start—the faster the better. Therefore, improving diagnostic accuracy and accelerating treatment initiation remain the key challenges for health systems to maximising benefits of reperfusion.

Accurate stroke diagnosis involves distinguishing between ischaemic lesions and intracerebral haemorrhage (ICH), a critical step in assessing patients’ eligibility for IVT. Additionally, ischaemic stroke must be differentiated from common mimics (eg, migraine, seizures, metabolic and functional disorders). Neuroimaging of acute stroke usually involves brain CT, which shows excellent accuracy in detecting ICH (~100%).^6^ However, high-field (HF) MRI currently yields the highest sensitivity and specificity in detecting acute ischaemic lesions. Specifically, diffusion-weighted imaging (DWI) becomes abnormal within few minutes of ischaemic stroke and has shown 86%–100% specificity and 73%–92% sensitivity (98% accuracy) in detecting early ischaemic lesions.^7^ Over the next hours, further ischaemic injury leads to vasogenic oedema, which is visible on fluid-attenuated inversion recovery (FLAIR) sequences. FLAIR offers a sensitivity of 90% in the acute phase and 88% in the subacute phase in detecting brain ischaemic changes.^8^

Advanced brain imaging modalities such as perfusion CT and perfusion-diffusion MRI can identify salvageable brain tissue, potentially extending the time window for reperfusion. Indeed, patients with viable brain tissue at CT perfusion or perfusion-diffusion MRI can benefit from IVT administered up to 9 hours of ischaemic stroke onset,^4^ and from EVT performed up to 24 hours (in patients with anterior circulation LVO).^9^ Additionally, MRI-guided IVT has been shown to significantly improve the functional outcome of patients with stroke of unknown onset who have an ischaemic lesion visible on DWI but no parenchymal hyperintensity on FLAIR.^10^

Despite these advantages, rapid access to HF-MRI is often limited by the presence of contraindications or by its unavailability, especially in small hospitals and at night.^3^ Indeed, installing 1.5T or 3T MRI scanners is expensive and very demanding for the infrastructure and it requires continuous maintenance, sophisticated siting and utilisation. A low-field (LF) MRI scanner could operate in a broader range of environments, such as the emergency department (ED) or the ambulance setting (mobile stroke unit, MSU). Very recently portable LF-MRI has been evaluated in critically ill patients and resulted able to detect several types of brain injury, including ischaemic stroke, ICH, subarachnoid haemorrhage, traumatic brain injury and brain tumours.^11^ A preliminary study conducted in the intensive care unit setting demonstrated also an acceptable mean examination time (about 35 min) to deliver a complete protocol of diagnostic-grade sequences (T1W, T2W, T2, FLAIR, DWI).^12^ As for ischaemic stroke, performing DWI at 64 mT (Hyperfine LF-MRI, Guilford, Connecticut, USA) has been shown to successfully identify early lesions.^11^,^13^ Furthermore, LF-MRI DWI and FLAIR sequences were able to detect infarcts as small as 4 mm in diameter in >90% patients across cortical, subcortical and cerebellar structures, showing a strong correlation between ischaemic volumes, stroke severity and functional outcomes at the discharge.^14^

Referring to ICH, LF-MRI was evaluated mostly in the subacute phase, therefore, data on the hyperacute assessment are largely missing.^15^ Interrater discordance among expert neuroradiologists was observed mainly in detecting small haematomas, especially located in the posterior fossa.^15^ Potential advantages and current limitation of LF-MRI in acute stroke are reported in box 1.

Box 1 **Applicability of low-field (LF) MRI in acute stroke: advantages and limitations**

**Potential advantages**

- LF-MRI provides point-of-care service to stroke patients.
- Well-maintained site, sophisticated setting and utilisation are not strictly necessary.
- The scanning protocol of LF-MRI is simpler compared with conventional high field (HF) MRI, thus potentially reducing the time of images acquisition.
- LF-MRI technology is compatible with nearby ferromagnetic materials and enables scanning outside the traditional MR suite. It is not necessary to remove vital sign monitors, intravenous infusion pumps or ventilators.
- LF-MRI allows easy patients handling and positioning and extends advanced neuroimaging applicability to complex patients (eg, critically ill patients, patients with agitation, accidental or implanted ferromagnetic devices such as pacemakers).
- Reduced staffing through easy data acquisition with automated stroke sequences (machine learning approach).
- Reduced patients transfer to different services, thus hampering risks associated with critical or complex patient mobilisation and probability of in-hospital infections.
- LF-MRI associated with an equipped van potentially may enable to diagnose and characterise acute stroke patients even in remote area or large city outskirts.
- Lower costs versus HF MRI.

**Limitations**

- No conclusive data are available on LF-MRI diagnostic accuracy, sensitivity and specificity in the acute stroke setting.
- Not known impact of implementing LF-MRI on door-to-needle time and time to intravenous thrombolysis initiation.
- Accuracy of LF-MRI in discriminating intracerebral haemorrhage from ischaemic stroke lesions must be improved to reach 100%, especially for small and subtentorial bleeds.
- Accurate identification of the infarct core and assessment of collateral flow must be implemented for guiding correct treatment choice.
- Integration between LF-MRI and tissue plasminogen activator administration for thrombolysis monitoring is still to be tested.
- LF-MRI was never investigated as a tool to guide treatment decisions in acute stroke care.
- No information is available on LF-MRI performance in assessing the evolution of ischaemic/haemorrhagic lesions over the chronic phase.
- Currently, only predetermined LF-MRI sequences are administrable, so that protocols that are more targeted on acute stroke need to be further developed and tested (eg, perfusion study, angiographic sequences).
- LF-MRI mobile stroke units have not been designed and validated in real clinical settings.

Here, we designed a multicentric open-label prospective trial to evaluate a point-of-care LF-MRI system in the diagnosis of acute stroke and in guiding treatment decisions.

## Methods and analysis

### Objectives

The objectives of the present study are the following: (1) To assess the diagnostic yield of LF-MRI for the diagnosis of acute stroke as compared with conventional neuroimaging (CT and HF-MRI). (2) To evaluate the performance of LF-MRI as a tool to guide treatment decisions in acute ischaemic stroke. This study will further develop LF-MRI technology and integrate it with artificial intelligence (AI) to facilitate interpretation of imaging findings. Further investigations of the present study include feasibility assessment of MSU equipped with LF-MRI and cost-effectiveness analysis of LF-MRI implementation in the acute stroke care.

### Study design

The Point-of-care low-field MRI in acute Stroke study is a multicentric, prospective clinical trial involving adult patients with suspected stroke. Recruitment of patients will be performed at three study units: Azienda Sanitaria Locale (ASL) Abruzzo 1, Hospitals of L’Aquila and Avezzano, Italy (ASLAQ); ASL Abruzzo 2, Hospital of Chieti, Italy (ASLCH); Istituto di Ricerca e Cura a Carattere Scientifico (IRCCS) Humanitas Research Hospital of Milan, Italy (HUM). Additional units contributing to the study will include:

- University of L’Aquila (UNIVAQ) will be in charge of study coordination, data analysis and management, optimisation of LF-MRI protocols, and cost-effectiveness assessment of LF-MRI within the Italian public care system.
- Marche Polytechnic University (UNIPM) will perform independent assessment and adjudication of anonymised clinical, LF-MRI and conventional neuroimaging data in a blinded fashion; it will also identify the best possible treatment option for patients with suspected ischaemic stroke (IVT, EVT, combined revascularisation treatment, no revascularisation).
- ‘G. Martino’ Polyclinic University Hospital (POLIME) will perform blinded and independent assessment and adjudication of anonymised clinical and conventional neuroimaging data, also will identify the best possible treatment option for patients with suspected ischaemic stroke and provide historical HF-MRI data of patients with stroke to be used for AI algorithms training.
- Free University of Bozen-Bolzano (UNIBZ) will develop and optimise AI algorithms suitable for LF-MRI images interpretation starting from anonymised conventional HF-MRI and LF-MRI data provided by ASLAQ, ASLCH, HUM and POLIME.
- University of Cassino and Southern Lazio (UNICAS) will perform a feasibility study to develop an ambulance (MSU) equipped with an LF-MRI scanner.

Agreement with conventional neuroimaging will be evaluated at different time points (hyperacute, acute—24 hours, subacute—72 hours, discharge, chronic—4 weeks) by ASLAQ, ASLCH, HUM, POLIPM and POLIME. At each time point, LF-MRI will be scheduled immediately after conventional neuroimaging to ensure comparability between performed investigations. In summary, this trial will provide necessary data to validate the use of LF-MRI in the acute stroke healthcare system.

Patients with suspected acute stroke will undergo diagnostic procedures and management according to usual clinical care. LF-MRI will be performed on admission, 24 hours, 72 hours, at the hospital discharge and 4 weeks after the event. LF-MRI findings will not be used to make clinical decisions on patients’ management. Every time a stroke dispatch is activated, the dedicated research staff will be alerted by the local neurologist on duty. The research staff will check eligibility criteria, obtain informed consent and perform study procedures independently of the personnel in charge of managing the acute stroke case. All included patients with suspected stroke will be managed and treated per usual care by the hospital staff not involved in study procedures. Conventional stroke imaging will be performed as needed according to current clinical practice (non-contrast CT+CT angiography and/or HF-MRI+MRI angiography in selected cases, with/without CT or HF-MRI perfusion study).

**Figure 1:**
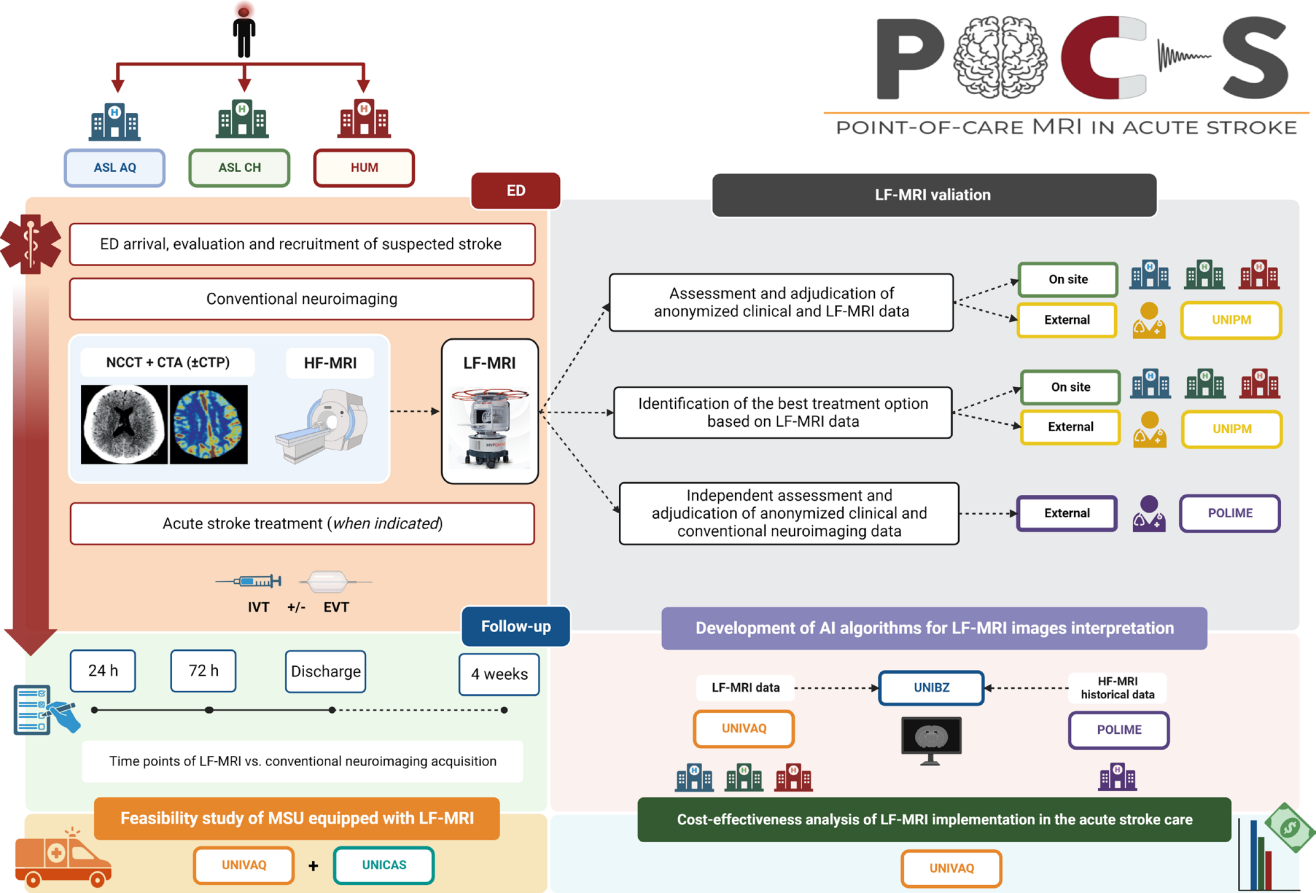
Study units and procedures of the POCS protocol. ASLAQ, ASL Abruzzo 1 (Hospitals of L’Aquila and Avezzano); ASLCH, ASL Abruzzo 2 (Hospital of Chieti); ED, emergency department; EVT, endovascular treatment; HF, high-field; HUM, Humanitas Research Hospital; IVT, intravenous thrombolysis; LF, low-field; MSU, mobile stroke unit; POCS, point-of-care low-field MRI in acute stroke; POLIME, ‘G. Martino’ Polyclinic University Hospital; UNIBZ, Free University of Bozen-Bolzano; UNICAS, University of Cassino and Southern Lazio; UNIPM, Marche Polytechnic University; UNIVAQ, University of L’Aquila.

For the aim of the study, all included patients will be investigated with LF-MRI at predefined time points (24 hours, 72 hours, hospital discharge, 4 weeks). The acquisition of LF-MRI will be performed by a dedicated study staff that will be distinct from the clinical staff involved in the care of the acute stroke patient, thus not subtracting time or resources to usual care. Information obtained by LF-MRI will not be used to take clinical decisions regarding further investigations or treatment. Due to feasibility reasons, the study will include only patients presenting to the ED with suspected stroke during weekdays daytime hours. Study procedures are summarised in figure 1.

### Study population, inclusion and exclusion criteria

The study will include consecutive patients admitted to the ED of recruiting centres with a dispatch of suspected stroke. Dispatches will be provided by Emergency Medical Services or ED physicians according to local acute stroke management protocols and current clinical practice.

Inclusion criteria are the followings: (1) symptoms suggestive of acute stroke (acute onset of a focal neurological deficit which can be referred to the involvement of a specific CNS region); (2) stroke onset <24 hours since when the patient was last known healthy; (3) age ≥18 years and (4) written informed consent provided by the patient himself or by proxy (for unconscious patients, cognitively impaired or aphasic).

Patients meeting at least one of the following criteria will be excluded from the study: (1) symptoms not indicative of acute stroke (eg, syncope, tonic or clonic activity, dizziness or wooziness alone, confusion and amnesia alone, subacute or chronic development of a focal neurological deficit); (2) inability to undergo LF-MRI due to critically impaired vital functions (eg, haemodynamically unstable patients, need of immediate life-saving manoeuvres); (3) implanted ferromagnetic devices potentially interfering with LF-MRI (eg, cochlear implants) and (4) impossibility to achieve written informed consent.

### LF-MRI equipment and imaging protocol

Three FDA and EU approved portable LF-MRI commercialised by Hyperfine Research (www.hyperfine.io) will be employed. All patients will be preliminarily tested with a metal detector to ensure the absence of potentially interfering ferromagnetic devices throughout the body. Patients will undergo LF-MRI directly in the CT suite during the downtime of preparation which usually occurs after urgent CT imaging (current mean door-to-imaging time: **~**20–25 min), thus not delaying the conventional diagnostic process and treatment administration. Specifically, LF-MRI acquisition will be performed during the time needed to obtain the neuroradiological report, waiting for results of essential laboratory tests, stroke nurse arrival, IVT initiation and/or preparation of the angiography room. For patients eligible for immediate IVT following brain CT, LF-MRI will be conducted simultaneously during the administration of IVT. In cases where acquisition of images with LF-MRI is supposed to delay initiation of revascularisation procedures, study procedures will be stopped and the patient will be excluded from the study. LF-MRI will also be repeated by the study staff at predefined time points: 24 hours, 72 hours, at the discharge and 4 weeks after the event.

**Figure 2:**
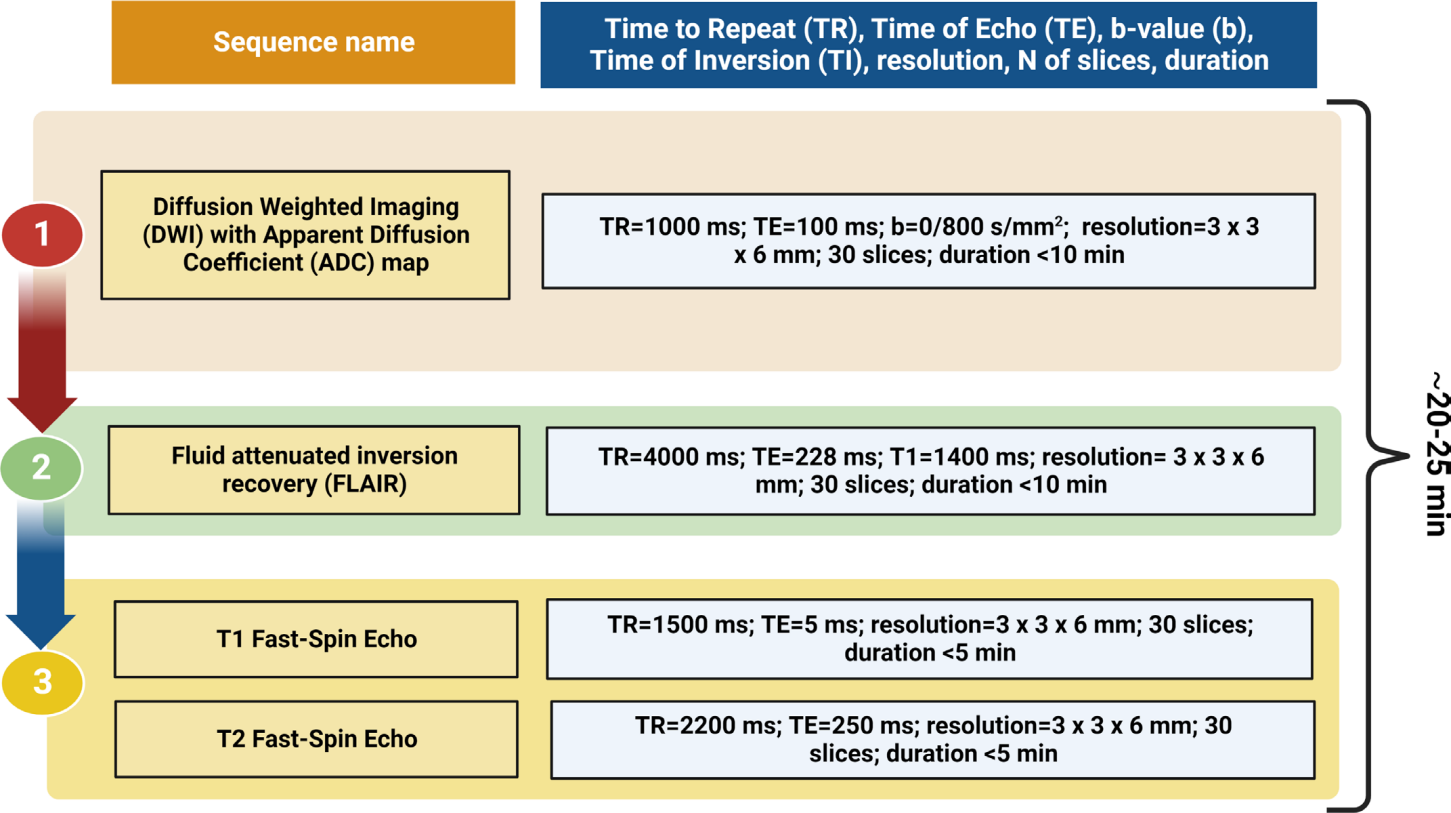
Prespecified low-field MRI minimum acquisition time pulse sequences protocol. DWI with ADC maps (1) will be acquired first to detect early stroke changes. Subsequently, FLAIR (2), T1 and T2 Fast-Spin Echo (3) sequences will be performed only when needed, according to clinical information (suspected stroke subtypes—eg, large vessel occlusion, potential treatment eligibility). The protocol is set up to have a total duration of ~20–25 min.

A prespecified acquisition of Fast-Spin Echo essential stroke sequences will be performed, starting with DWI, apparent diffusion coefficient (ADC) mapping and FLAIR. Other sequences will be performed based on DWI (ADC) and FLAIR findings and on clinical information (suspected stroke subtypes, eligibility to reperfusion treatment), to shorten the duration of LF-MRI acquisition and avoid treatment delay. The prespecified LF-MRI minimum acquisition time pulse sequences protocol is displayed in figure 2. Furthermore, an angiographic sequence will be tested to assess the presence of cerebral LVO or stenosis. This sequence will evaluate the loss of signal due to blood flow in the time frame between the excitation pulse and signal refocusing. Overall, LF-MRI acquisition has been set up to require ~20–25 min to complete, as estimated using the Hyperfine model of portable LF-MRI.

### Time points and collected variables

The following clinical variables will be collected at the specified time point:

1. Hyperacute (ED): demographical data, time from symptom onset (precise onset, unwitnessed, wake-up stroke), onset-to-door-time, door-to-imaging time, clinical severity (National Institute of Health Stroke Scale, NIHSS), type of neuroimaging, duration of LF-MRI scanning, conventional neuroimaging and LF-MRI findings (detection of ischaemic or haemorrhagic lesion(s), lesion(s) number, location and volume, detection and characteristics of possible LVO, collateral flow assessment, Alberta Stroke Programme early CT score^16^ and/or DWI/FLAIR mismatch for MR, pre-EVT grade of perfusion according to the thrombolysis in cerebral infarction—TICI—scale), treatment decision (IVT, EVT, IVT+EVT, none), time (door-to-needle for IVT and door-to-groin for EVT), clinical outcome (NIHSS score immediately after IVT/EVT—if performed) and reperfusion outcome (TICI score immediately after EVT—if performed).
2. Acute (24 hours) and subacute (72 hours): clinical severity (NIHSS), conventional neuroimaging type and findings, LF-MRI findings (lesion volume and characteristics, evolution—for example, presence and entity of haemorrhagic transformation).
3. Hospital discharge: days of hospital stay, functional outcome at discharge (modified Rankin scale), final adjudication of the event (ischaemic stroke, ICH, SAH or mimic), assessment of aetiology according to the Trial of ORG 10172 in Acute Stroke Treatment (TOAST) classification^17^—for ischaemic stroke.
4. Chronic (4 weeks): conventional neuroimaging type and findings, LF-MRI findings (lesion volume, characteristics and evolution), assessment of stroke aetiology (TOAST)^17^ also considering findings from postdischarge clinical investigations.

### Outcomes measurement

All events will be adjudicated as ischaemic stroke, ICH, SAH or stroke mimic. For each of the three recruiting units a senior neurologist and a neuroradiologist will adjudicate clinical data and neuroimaging findings at LF-MRI. A different pair of senior neurologist and neuroradiologist will adjudicate events based on clinical data and conventional neuroimaging. The pair assessing LF-MRI will be blinded to findings of conventional neuroimaging and vice-versa. All included patients will be independently adjudicated for variables of interest by the study staff of UNIPM or POLIME: UNIPM will adjudicate clinical and LF-MRI findings; POLIME will adjudicate clinical and conventional neuroimaging findings. In case of disagreements, final adjudication will be performed by the principal investigator of the study. UNIPM will identify the best possible treatment option for patients with ischaemic stroke based on LF-MRI, while POLIME will identify treatment options based on conventional imaging.

This study has a primary outcome and a number of cosecondary outcomes. The primary outcome measure of the study is the accuracy of LF-MRI in the diagnosis of stroke in the acute phase (endpoint: sensitivity and specificity with respect to final adjudication of the event). Secondary outcome measures include:

1. Detection of lesions at LF-MRI versus non-contrast CT: to compare the accuracy of LF-MRI scans with the accuracy of routinely performed head CT scans for infarcts and haemorrhage detection (endpoint: agreement between LF-MRI and head CT scans in detecting infarcts and haemorrhages).
2. Detection of lesions at LF-MRI versus HF-MRI: to compare the accuracy of LF-MRI scans with the accuracy of routinely performed brain HF-MRI scans for infarcts and haemorrhage detection (endpoint: agreement between LF-MRI and HF-MRI scans in detecting infarcts and haemorrhages).
3. Treatment decision-making based on LF-MRI: to evaluate the impact of LF-MRI on the clinical decision-making process related to revascularisation treatments for ischaemic stroke, as compared with the conventional acute stroke diagnostic (endpoint: agreement between the best treatment option based on LF-MRI and the best treatment option based on conventional imaging).
4. Impact of LF-MRI implementation on the door-to-needle time (DTNT) in patients eligible to IVT: to evaluate whether the implementation of LF-MRI may impact on the DTNT in patients treated with IVT at each recruiting site (endpoint: comparison between the mean DTNT recorded during the 2 months before the protocol initiation—and in patients who refuse to undergo LF-MRI during the study period—and the mean DTNT estimated basing on the results of LF-MRI).
5. Early identification of stroke mimics with LF-MRI: to assess whether LF-MRI may improve the detection of patients with stroke mimics in the acute phase and the discrimination with acute ischaemic stroke (endpoint: agreement between LF-MRI and conventional imaging in detecting stroke mimics).
6. Agreement between LF-MRI and conventional brain imaging at different post-stroke time points: to assess the performance of LF-MRI at different phases of the ischaemic stroke process (hyperacute, acute—24 hours, subacute—72 hours and chronic—4 weeks from the event) and to compare with findings from conventional neuroimaging (endpoint: agreement between LF-MRI and conventional neuroimaging at different stroke phases).

### Further investigations

The study will also aim to address the following points:

1. Identification of an optimal LF-MRI scanning protocol for acute stroke: to identify the optimal LF-MRI scanning protocol that can maximise the accuracy of ischaemic/haemorrhagic stroke lesions detection without lengthening the overall time of neuroimaging.
2. Cost-effectiveness analysis of LF-MRI implementation: to perform an analysis of direct and indirect costs associated with the implementation of LF-MRI in the emergency setting. Direct and indirect costs related to the proposed technological and organisational innovations will be analysed by experts at UNIVAQ (Economics Department), thus pointing out potential budgetary implications at either individual ASLs/hospitals or at regional level. Subsequently, impacts on the organisational pathway and human resource management resulting from LF-MRI implementation will also be assessed.
3. Development of AI-based algorithm for LF-MRI: an AI system for LF-MRI images processing based on machine learning will be developed by experts at UNIBZ (Faculty of Computer Science). The software will be refined through the automated analysis of HF-MRI images that will be downgraded to a lower resolution comparable to LF-MRI. A large set of gold standard HF (1.5 T and 3 T) MRI acquisitions of patients with acute stroke will be provided by a participating centre (POLIME).
4. Feasibility study of LF-MRI implementation on MSU: a comprehensive evaluation of technical and safety issues associated with the implementation of LF-MRI technology on MSU will be carried out by experts of UNICAS (Civil and Mechanical Engineering Department) in collaboration with MRI hardware experts at UNIVAQ (Department of Life, Health & Environmental Sciences).

### Sample size calculation

The sample size of this study has been calculated considering the sensitivity and specificity of conventional HF-MRI (91%–100% and 86%–100%, respectively), available data on LF-MRI diagnostic performance (∼90% sensibility and ∼80% specificity),^11^,^13^ as well as the estimated prevalence of stroke among all patients admitted to the ED with an Emergency Service dispatch of suspected stroke (∼80%)^2^ based on a 20% prevalence of stroke mimics.^18^ We calculated a minimum sample size of n=273 patients to obtain a reliable estimate of LF-MRI diagnostic performance (95% CI, 1−β=80%) and to compare with conventional neuroimaging. Referring to the evaluation of agreement between LF-MRI and conventional neuroimaging (Cohen kappa coefficient expected value=0.8, expected outcome ∼20%, 1−β=80%), we calculated a minimum sample size of n=214 patients. All our estimate take into account a rate of ∼10% patients potentially dropping out from the study. Therefore, to maximise the accuracy of our analyses, we aim to include an overall population of 100 patients for each recruiting centre (300 patients in total).

### Statistical plan

Categorical variables will be presented as frequency and percentage (%). Continuous variables will be presented as mean (±SD) or median (IQR), according to their distribution. The Shapiro-Wilk test will be performed to assess the normality of variables. Comparison between categorical variables will be performed using the Pearson χ^2^ test, while continuous variables will be compared using the Student’s t-test or the analysis of variance test depending on group numerosity. For comparison including more than two groups, a post hoc analysis will be performed. Initially, a comprehensive description of LF-MRI findings will be provided. LF-MRI diagnostic accuracy will be assessed as sensitivity and specificity, which in turn will be calculated based on the proportion of true/false positives and true/false negatives in respect to the gold standard (ie, the final adjudication of the event, based on clinical and radiological examinations). Positive and negative predictive values will also be calculated. Agreement between LF-MRI and conventional neuroimaging will be evaluated using the Cohen kappa coefficient. The statistical significance will be set at p<0.05.

### Study period

The start date of the study was 13 February 2023, and the expected end data is 12 February 2027. The enrolment period is 3 years, from 1 February 2024 to 31 January 2027.

### Data collection and management

All patients enrolled will provide written informed consent before their data are included in the study. All data, including neuroimaging findings, will be anonymised by using code numbers. All clinical data will be collected and stored using a Research Electronic Data Capture (REDCap) predefined Case Report Form (CRF) on a secure server hosted by UNIVAQ. Corresponding medical evaluations and adjudication of treatment decisions will be signed with name and date by the rating researcher. Data quality control measures will include queries to identify missing data, outliers and discrepancies. Only researchers will be allowed to view the REDCap CRF. If a researcher withdraws from the study, the records are handed over to a mutually designated investigator. The original medical records will be kept as complete as the original documents of clinical trials. All data and images transfers among participating centres will be done via secure, authenticated and encrypted HTTPS connections.

### Protocol amendments

Amendments to the protocol will be made according to Good Clinical Practice requirements and must be approved by the funder and by the ethics committees of all participating centres before being applied.

### Data monitoring

This study is a multicentric prospective open-label trial without significant safety problems. An external data monitoring committee is not required. The principal investigator (SS) and study staff will monitor data internally and will meet in person or by phone on a weekly basis to ensure the study is proceeding as intended.
