# Supplementary material for: Point-Of-Care low-field MRI in acute Stroke (POCS): protocol for a multicentric prospective open-label study evaluating diagnostic accuracy
Source: BMJ Open. 2024 Jan 31;14(1):e075614. doi: 10.1136/bmjopen-2023-075614 (PMC10831427; doi:10.1136/bmjopen-2023-075614)
Supplement: Supplementary file 1 [file bmjopen-2023-075614.reviewer_comments.pdf]

PEER REVIEW HISTORY

BMJ Open publishes all reviews undertaken for accepted manuscripts. Reviewers are asked to complete a checklist review form (**http://bmjopen.bmj.com/site/about/resources/checklist.pdf**) and are provided with free text boxes to elaborate on their assessment. These free text comments are reproduced below.

ARTICLE DETAILS

**TITLE (PROVISIONAL)**	Point-Of-Care low-field MRI in acute Stroke (POCS): protocol for a multicentric prospective open label study evaluating diagnostic accuracy
**AUTHORS**	Foschi, Matteo; Galante, Angelo; Ornello, Raffaele; Necozione, Stefano; Marini, Carmine; Muselli, Mario; Achard, Paola Olimpia; Fratocchi, Luciano; Vinci, Sergio; Cavallaro, Marco; Silvestrini, Mauro; Polonara, Gabriele; Marcheselli, Simona; Straffi, Laura; Colasurdo, Marco; Sorrentino, Luca; Franconi, Enrico; Alecci, Marcello; Caulo, Massimo; Sacco, Simona

VERSION 1 – REVIEW

**REVIEWER**	Buck, Brian H.<br>Univ Alberta, Medicine
**REVIEW RETURNED**	02-Aug-2023

**GENERAL COMMENTS**	The manuscript is a protocol for the Point-Of-Care low-field MRI in acute Stroke (POCS) study. The study is an ongoing multicentric prospective open-label study evaluating the diagnostic accuracy of low-field MRI in the evaluation of patients with acute suspected stroke at three sites in Italy. Overall the protocol is well-designed and the study is examining a highly novel approach to imaging acute stroke.<br><br>I have the following comments:<br><br>1) pg 1, line 28. The authors state that IVT can be used in selected patients “with favourable brain perfusion imaging….”. The authors have referenced the WAKE-UP trial which looked at MRI FLAIR DWI mismatch. This should be discussed further especially since the study involves MRI acutely.<br><br>2) Page 1, line 45 states: “MRI yields the highest sensitivity….”. This should be qualified that this is for early ischemic stroke, not hemorrhage. The article cited is a review article that does not discuss MRI sensitivity/specificity. I would cite original references rather than a review article for this important statement as this provides the rationale for the study.<br><br>I would also add to the discussion the high sensitivity of CT to detect hemorrhage (as per the citation almost 100%) as this is relevant when making early thrombolysis decisions.<br><br>3) HF-MRI and LF-MRI should be defined at the first usage on line 51 (page 1)

	4) There is a discrepancy between time points on line 5, page 9 and those on the prior page. Should this include “hyperacute”?<br><br>5) On page 9, There is a statement that LF-MRI “will be performed during the time needed to obtain the neuroradiological report etc…..” and that treatment will not be delayed,<br><br>The protocol should report the study sites' typical DTN and not just time to CT to understand how the LF-MRI would fit into the workflow at study sites without delaying reperfusion therapy which is the main risk of implementing this study protocol.<br><br>Can you expand the on how it will be possible to add 20-25 minutes of study imaging without delaying thrombolysis? Many sites globally administer IVT within minutes of completion of the NCCT and often while the patient is in the scanner. The protocol states the LF-MRI acquisition takes 20-25 minutes, plus patients will need to be moved to the scanner.<br><br>Does the study have a process for monitoring the impact of the study on DTN? Specifically, as a safety variable could you look at DTN study patients vs those not enrolled? This would help plan future trials.<br><br><br>6) Are the LF-MRI units funded by the grant or provided in kind by the manufacturer? If the latter, this should be included in the funding section.<br><br>7) Figure 1 states that conventional neuroimaging is CT+CTA, the picture shows a CT and CTP. I would suggest changing to CT+CTA+-CTP (or something similar)

**REVIEWER**	Donnan, Geoffrey A<br>Natl Stroke Inst Res
**REVIEW RETURNED**	20-Nov-2023

**GENERAL COMMENTS**	A most interesting protocol describing the validation of a novel light weight imaging device, MR based. Overall the protocol reads very well with only a few relatively minor issues to be addressed which would improve the manuscript:<br><br>1. Include the proposed sample size in the abstract<br>2. On page 6 – Mobile stroke unit plans - what does “without the need to deal with patient data” mean? Does this mean that patients will not be imaged and modelling only will take place?<br>3. It is not clear what the projected time delay between standard imaging and LF-MRI is likely to be. If too long, interpretation of comparative images will be difficult. Please clarify.<br>4. There are numerous grammatical issues which need to be addressed. This is completely understandable given that English is not the authors' first language. This can be addressed separately by the editorial staff

VERSION 1 – AUTHOR RESPONSE

Reviewer: 1
Dr. Brian H. Buck, Univ Alberta
Comments to the Author:
The manuscript is a protocol for the Point-Of-Care low-field MRI in acute Stroke (POCS) study. The study is an ongoing multicentric prospective open-label study evaluating the diagnostic accuracy of low-field MRI in the evaluation of patients with acute suspected stroke at three sites in Italy. Overall the protocol is well-designed and the study is examining a highly novel approach to imaging acute stroke.
REPLY: we thank the Reviewer for the overall positive evaluation of the protocol and for his valuable suggestions.

I have the following comments:

1) pg 1, line 28. The authors state that IVT can be used in selected patients “with favourable brain perfusion imaging….”. The authors have referenced the WAKE-UP trial which looked at MRI FLAIR DWI mismatch. This should be discussed further especially since the study involves MRI acutely.
REPLY: we thank the Reviewer for this suggestion. We added a paragraph to explain the importance of advanced neuroimaging techniques for extending the eligibility to acute reperfusion therapies in the paragraph of the introduction on neuroimaging in acute stroke:
“Advanced brain imaging modalities such as perfusion CT and perfusion-diffusion MRI can identify salvageable brain tissue, potentially extending the time window for reperfusion. Indeed, patients with viable brain tissue at CT perfusion or perfusion-diffusion MRI can benefit from IVT administered up to 9 hours of ischemic stroke onset, [4] and from EVT performed up to 24 hours (in patients with anterior circulation LVO). [9] Additionally, MRI-guided IVT has been shown to significantly improve the functional outcome of patients with stroke of unknown onset who have an ischemic lesion visible on DWI but no parenchymal hyperintensity on FLAIR. [10]“.

2) Page 1, line 45 states: “MRI yields the highest sensitivity….”. This should be qualified that this is for early ischemic stroke, not hemorrhage. The article cited is a review article that does not discuss MRI sensitivity/specificity. I would cite original references rather than a review article for this important statement as this provides the rationale for the study.
REPLY: We rephrased the text on MRI sensitivity by specifying the diagnostic performance of MRI in the diagnosis of acute ischemic lesions at different stages and provided original references as suggested by the Reviewer.
“However, high-field (HF) magnetic resonance imaging (MRI) currently yields the highest sensitivity and specificity in detecting acute ischemic lesions. Specifically, DWI becomes abnormal within few minutes of ischemic stroke and has shown 86-100% specificity and 73-92% sensitivity (98% accuracy) in detecting early ischemic lesions. [7] Over the next hours, further ischemic injury leads to vasogenic edema, which is visible on fluid attenuated recovery (FLAIR) sequences. FLAIR offers a sensitivity of 90% in the acute phase and 88% in the subacute phase in detecting brain ischemic changes. [8]”
I would also add to the discussion the high sensitivity of CT to detect hemorrhage (as per the citation almost 100%) as this is relevant when making early thrombolysis decisions.
REPLY: We specified the excellent diagnostic accuracy of brain NCCT in detecting ICH and mentioned the importance to evaluate patients’ eligibility to acute reperfusion treatments at the beginning of the paragraph on neuroimaging (introduction):
“Accurate stroke diagnosis involves distinguishing between ischemic lesions and intracerebral hemorrhage (ICH), a critical step in assessing patients' eligibility for IVT. Additionally, ischemic stroke must be differentiated from common mimics (e.g., migraine, seizures, metabolic and functional

disorders). Neuroimaging of acute stroke usually involves brain computed tomography (CT), which shows excellent accuracy in detecting ICH (~100%). [6]"

3) HF-MRI and LF-MRI should be defined at the first usage on line 51 (page 1)
REPLY: we defined HF- and LF- acronyms at their first usage.

4) There is a discrepancy between time points on line 5, page 9 and those on the prior page. Should this include "hyperacute"?
REPLY: we added hyperacute to time points for agreement evaluation in page 9.

5) On page 9, There is a statement that LF-MRI "will be performed during the time needed to obtain the neuroradiological report etc….." and that treatment will not be delayed,

The protocol should report the study sites' typical DTN and not just time to CT to understand how the LF-MRI would fit into the workflow at study sites without delaying reperfusion therapy which is the main risk of implementing this study protocol.
REPLY: we thank the Reviewer for pointing out this important issue. As historical data on DTNT could significantly differ from current timing due to recent improvement in the acute stroke procedure (e.g., implementation of IVT administration directly into the CT suite), we decided to perform a prospective evaluation of DTNT for IVT-eligible patients at each recruiting site during the 2 months before initiation of the POCS study, as well as in patients who refuse to undergo LF-MRI. Thereafter, we will compare the estimated DTNT based on results of LF-MRI in respect to the recorded mean DTNT. We added this evaluation into the section "outcomes measurements" in Page 9:
4) impact of LF-MRI implementation on the door-to-needle time (DTNT) in patients eligible to IVT: to evaluate whether the implementation of LF-MRI may impact on the DTNT in patients treated with IVT at each recruiting site (endpoint: comparison between the mean DTNT recorded during the 2 months before the protocol initiation - and in patients who refuse to undergo LF-MRI during the study period - and the mean DTNT estimated basing on the results of LF-MRI).

Can you expand the on how it will be possible to add 20-25 minutes of study imaging without delaying thrombolysis? Many sites globally administer IVT within minutes of completion of the NCCT and often while the patient is in the scanner. The protocol states the LF-MRI acquisition takes 20-25 minutes, plus patients will need to be moved to the scanner.
REPLY: to avoid time delay we planned to place LF-MRI directly in the CT suite and to perform it immediately after the end of conventional neuroimaging. LF-MRI will be performed during the time required to complete the CT report while waiting for essential blood tests results or during the time needed to obtain informed consent for revascularization procedures. In the case of patients with clear eligibility to IVT and who don't need to wait for the results of further investigations (beyond brain CT), we will perform LF-MRI during IVT administration. We clarified in the text as follows (Page 8):
"Patients will undergo LF-MRI directly in the CT suite during the downtime of preparation which usually occurs after urgent CT imaging (current mean door-to-imaging time: ~20-25 minutes), thus not delaying the conventional diagnostic process and treatment administration. Specifically, LF-MRI acquisition will be performed during the time needed to obtain the neuroradiological report, waiting for results of essential laboratory tests, stroke nurse arrival, IVT initiation and/or preparation of the angiography room. For patients eligible for immediate IVT following brain CT, LF-MRI will be conducted simultaneously during the administration of IVT."

Does the study have a process for monitoring the impact of the study on DTN? Specifically, as a safety variable could you look at DTN study patients vs those not enrolled? This would help plan future trials.

REPLY: As mentioned above, the impact of LF-MRI on DTNT will be assessed by comparing the

mean DTNT recorded in the 2 months preceding the protocol implementation at each site (as well as in patients who refuse to undergo LF-MRI) with the mean DTNT estimated during the study period basing on results of LF-MRI.
We anticipate that we planned to perform, after validation, an alternating-week, cluster-controlled study on the model of the MSU landmark trial (Grotta GC et al., NEJM, 2021) to better understand the effects of LF-MRI on safety variables, including DTNT.

6) Are the LF-MRI units funded by the grant or provided in kind by the manufacturer? If the latter, this should be included in the funding section.
REPLY: costs for LF-MRI devices will be totally covered by the grant funding. We added a statement in the funding section as follows:
"The purchase of the LF-MRI devices will be totally covered by grant funds."

7) Figure 1 states that conventional neuroimaging is CT+CTA, the picture shows a CT and CTP. I would suggest changing to CT+CTA+-CTP (or something similar)

REPLY: we added (±CTP) to the picture, as suggested. This was also specified within the text when mentioning conventional neuroimaging exams.

Reviewer: 2
Dr. Geoffrey A Donnan, Natl Stroke Inst Res
Comments to the Author:
A most interesting protocol describing the validation of a novel light weight imaging device, MR based. Overall the protocol reads very well with only a few relatively minor issues to be addressed which would improve the manuscript:
REPLY: we thank the Reviewer for recognizing the positive qualities of our study protocol and for his very useful observations.

1. Include the proposed sample size in the abstract
REPLY: we added the estimated sample size into the abstract ("The estimated sample size is 300 patients").

2. On page 6 – Mobile stroke unit plans - what does "without the need to deal with patient data" mean? Does this mean that patients will not be imaged and modelling only will take place?
REPLY: the sentence indicates that for the MSU modelling, UNICAS will not require direct access to clinical data acquired from patients during the LF-MRI validation. As this specification is not relevant and may create confusion, we decided to delete it.

3. It is not clear what the projected time delay between standard imaging and LF-MRI is likely to be. If too long, interpretation of comparative images will be difficult. Please clarify.
REPLY: at each time point, LF-MRI will be performed immediately after neuroimaging, thus avoiding delay between the 2 exams. We specified in the text as follows (Page 6):
"At each time point LF-MRI will be scheduled immediately after conventional neuroimaging to ensure comparability between performed investigations."

4. There are numerous grammatical issues which need to be addressed. This is completely understandable given that English is not the authors' first language. This can be addressed separately by the editorial staff
REPLY: we performed accurate proof-reading and corrected all grammar inaccuracies that we were able to identify.

Lastly, we updated details on the enrollment period as we are still finalizing the purchase procedure

for the LF-MRI devices.
“The enrollment period is 3 years, from February 1st 2024, to January 31st 2027.”

VERSION 2 – REVIEW

**REVIEWER**	Buck, Brian H.<br>Univ Alberta, Medicine
**REVIEW RETURNED**	31-Dec-2023

**GENERAL COMMENTS**	The authors have adequately addressed my comments and appropriately revised the manuscript. I have no further comments.
